# Evaluating a Large-Scale Multi-Center Outreach Program for Appointment Booking and Patient Engagement: A Quality Improvement Study

**DOI:** 10.7759/cureus.102480

**Published:** 2026-01-28

**Authors:** Rabih Abou Leila, Mohammad Albanna

**Affiliations:** 1 Family Medicine, American Mission Hospital, Manama, BHR; 2 Nursing, International Medical Center, Jeddah, SAU

**Keywords:** access to health care, care coordination, continuity of patient care, healthcare operations, prevention strategies

## Abstract

Introduction

Missed appointments (no-shows) and lapses in follow-up care are common challenges in healthcare, adversely affecting outcomes and efficiency. This study evaluated a system-wide quality improvement initiative implementing a centralized outbound call program aimed at improving appointment scheduling and continuity of care across a tertiary hospital and its satellite clinics.

Methods

The intervention (May to November 2025) consisted of a centralized call-center team that conducted 548,089 outbound calls to patients for three campaign types: (1) *administrative recovery* (rescheduling missed/canceled visits), (2) *continuity of care* (post-visit, emergency department, or post-discharge follow-ups), and (3) *preventive/safety* outreach (e.g., preventive screenings, abnormal result follow-ups). Callers used standard scripts and attempted up to three calls per patient as needed. Outcomes were analyzed retrospectively at the call-attempt level. The primary outcome was successful appointment booking (new appointment scheduled during/immediately after the call). The secondary outcome was patient engagement, defined as either a booked appointment or confirmation that an appropriate follow-up was already in place. Key outcomes were compared between the main hospital and satellite clinics using chi-square tests. The study was classified as a quality improvement activity and exempt from IRB review.

Results

Out of 548,089 call attempts, 66,238 new appointments were booked (12.1% of all calls). After excluding wrong numbers, calls about deceased patients, and calls to patients who already had an upcoming appointment, the adjusted booking rate was 14.6%. An additional 90,774 calls (16.6%) confirmed that patients already had a scheduled follow-up. Combining these, about 28.6% of all calls resulted in verified engagement with care (either new or pre-existing). Conversely, 42.9% of calls reached patients who declined scheduling, and 25.7% of calls did not reach the patient (no answer or voicemail). Booking outcomes were similar at the main hospital (12.3%) and clinics (11.4%, *p*<0.001), but overall engagement was higher for hospital calls (31.0% vs. 20.7%, *p*<0.001) due to more patients already having follow-ups. Campaign-level results varied: preventive screening calls yielded low booking rates (e.g., ~5% for mammography outreach) with many declines, whereas follow-up on abnormal results achieved higher booking rates (~26%) and often found existing appointments. A chi-square test confirmed significant heterogeneity in engagement across campaign types (*p*<0.001). No intervention-related adverse events were identified.

Conclusion

Implementing a centralized outreach program across multiple centers was associated with improved appointment connectivity, scheduling over 66,000 appointments and verifying care plans for an additional 90,000 patients within seven months. The initiative’s impact varied by context-patients from the main hospital were often already in care, while those from satellite clinics benefited more from new scheduling. These findings underscore the importance of tailoring outreach strategies to patient needs and clinical context. The program demonstrates a feasible, scalable approach to reducing no-shows and enhancing continuity of care. Future efforts should focus on increasing patient contact rates (e.g., via multimodal communication), understanding reasons for declined outreach, and assessing downstream clinical outcomes.

## Introduction

Missed appointments, delayed follow-up, and underuse of preventive services remain persistent system-level challenges in healthcare delivery, contributing to inefficiency, avoidable patient harm, and increased costs. Systematic reviews consistently demonstrate that reminder and outreach interventions improve appointment attendance, with live telephone outreach outperforming automated reminders, particularly among patients at higher risk of non-attendance and disengagement [1-3].

Beyond appointment adherence, failures in completing follow-up for diagnostic results, referrals, and post-discharge care are well-recognized contributors to diagnostic delay and preventable harm. Contemporary patient safety literature emphasizes the importance of closed-loop communication systems that not only initiate follow-up but also verify completion, confirm patient understanding, and identify barriers or disengagement when recommended actions are not completed [4-6]. Recent systematic reviews estimate that communication failures contribute to 13-24% of patient safety incidents, underscoring the safety relevance of outreach activities that actively confirm follow-up status rather than relying solely on passive notification [6].

Preventive outreach presents additional complexity. Although outreach initiatives can improve screening uptake, randomized trials and systematic reviews consistently report lower immediate booking conversion compared with administrative recovery or post-encounter follow-up. These differences reflect behavioral, perceptual, and motivational barriers rather than access limitations alone and highlight the limitations of booking-only outcomes as indicators of outreach performance [7-9].

Despite a growing evidence base, most published evaluations of outreach interventions remain limited to single clinical contexts, isolated campaign types, or individual care settings. There is limited literature examining centralized outreach as a system-wide quality improvement intervention, comparing performance across multiple care settings and outreach intents, or explicitly evaluating outreach as a mechanism for engagement detection - defined as confirmation of follow-up completion, identification of disengagement, or recognition of barriers preventing action - alongside appointment booking. To address this gap, our healthcare network implemented a centralized, multi-center outbound telephone outreach program across a tertiary hospital and its affiliated ambulatory clinics. This study reports the design, implementation, and outcomes of this quality improvement initiative using SQUIRE 2.0 guidelines, with successful appointment booking defined as the primary outcome and engagement detection as a secondary, safety-relevant outcome.

## Materials and methods

Setting and intervention

This study was conducted in a multi-center healthcare network comprising a large tertiary care hospital and several affiliated outpatient clinics. In early 2025, the health system established a centralized call center team to perform proactive patient outreach across all sites. Outreach operations were centralized to ensure consistency: a single trained team used standardized workflows and scripts for all calls, regardless of which clinic or hospital service the patient attended.

Intervention description

The outbound call program targeted patients for follow-up or rescheduling based on predefined campaigns: (1) administrative recovery (contacting patients who missed or canceled appointments to promptly reschedule them), (2) continuity of care (following up after clinical encounters, including post-discharge calls [after hospital inpatient stays], post-ED-visit calls, and follow-ups after specialist or primary care outpatient visits, to ensure appropriate next steps were scheduled), (3) preventive/safety follow-up (reaching out for preventive health measures, such as cancer screenings that were due or overdue, and ensuring follow-up of important test results, such as abnormal lab or imaging results that required an appointment but might not have been booked).

Patients eligible for each campaign were identified through the electronic health record (EHR) and scheduling systems. For example, daily lists of patients who had no-shows or canceled appointments were generated for the recovery campaign, and lists of recently discharged patients without a follow-up visit were generated for transitional care outreach. Preventive outreach lists were drawn from health maintenance registries (e.g., women overdue for mammograms).

A team of call center staff (trained in customer service and basic medical appointment triage) conducted the calls. Standardized scripts were developed for each campaign type to guide the conversation. These scripts included verification of patient identity, an explanation of the call’s purpose, and tailored messaging (for instance, emphasizing the importance of follow-up care or preventive screening). Staff could directly schedule appointments during the call through the shared scheduling system. Each patient was typically called up to three times on different days/times if prior attempts were unsuccessful, unless a definitive outcome (such as reaching the patient) was achieved sooner. Voicemails were left on the first attempt, if possible, with a callback number provided.

Throughout the intervention period (May 1, 2025, to November 30, 2025), the program was implemented as part of routine operations. Importantly, this was a pragmatic quality improvement initiative rather than a research experiment; all eligible patients received the intervention (outreach calls) as part of standard care processes, without a control group of patients withheld from outreach.

Study design and data collection

We performed a retrospective observational evaluation of the call program’s outcomes. All call attempts made by the outreach team during the seven-month period were logged in a centralized database with their final disposition. Because some patients required multiple call attempts (e.g., if not reached on first try), the unit of analysis was defined as a single call attempt, each with one final outcome recorded.

For each call attempt, the following outcome categories were defined and captured: (1) booked appointment (the patient agreed to and scheduled a new appointment during or immediately after the call), (2) already had appointment (the patient indicated that a follow-up appointment was already scheduled or a plan was in place, e.g., “I already have an appointment next week” or “My doctor told me to come back in 3 months and I will”), (3) declined scheduling (the patient was reached but explicitly declined to book an appointment, which includes deferring indefinitely or refusing the offered service), (4) no response/voicemail (the call attempt did not reach the patient ;no answer or reached voicemail without a definitive response), (5) no slot available (the patient was willing to schedule but no appointment slot was available during the call; this was tracked to identify capacity issues), (6) wrong number (the contact phone number was incorrect or not in service), (7) deceased (the patient was found to be deceased; this information was updated in records during the call attempt).

Each call attempt was thus categorized into one of these mutually exclusive final dispositions by the calling staff using a standardized logging interface.

Main outcome metrics

Two main outcome metrics were defined for analysis:

Successful appointment booking (primary outcome): a call resulting in a new appointment scheduled (i.e., “booked appointment” outcome).

Patient engagement (secondary outcome): a call resulting in the patient either booking a new appointment or confirming that appropriate follow-up was already in place (either outcome “booked appointment” or “already had appointment”). This metric was intended to capture any call that achieved continuity of care, whether by scheduling a needed visit or verifying that the patient did not require scheduling because they were already engaged in care.

For analytical purposes, we distinguished between “operationally eligible” calls for booking and calls that included patients with existing appointments. Specifically, calls to wrong numbers or deceased patients were considered ineligible for any outcome and were excluded from certain rate calculations. Likewise, for calculating the appointment booking rate, calls where the patient already had an appointment were excluded from the denominator since no new booking was needed or possible in those cases (we term this the adjusted booking rate). However, for calculating engagement rates, those calls were included because confirming an existing appointment was considered a positive engagement outcome.

We also categorized calls by care setting (calls pertaining to follow-up at the main hospital vs. at satellite clinics). Each call log entry contained the clinic or service context, allowing us to aggregate outcomes for calls related to the tertiary hospital services versus those related to the satellite ambulatory clinics. This comparison was of interest to determine if the outreach program performed differently in the centralized hospital setting compared to community-based clinics.

Analysis

We summarized overall call outcomes using descriptive statistics. The primary outcome (proportion of calls resulting in a booked appointment) was calculated in two ways: crude rate (using all call attempts as the denominator) and adjusted rate (excluding calls that were not actionable for booking, i.e., wrong number, deceased, or already had appointment). The secondary outcome (engagement rate) was calculated as the proportion of call attempts that led to either a booking or confirmation of existing follow-up, using all calls as the denominator (since engagement by definition includes those who already had appointments).

To assess differences by setting, we compared outcome distributions for calls related to the main hospital versus satellite clinics. Chi-square tests of independence were used to evaluate whether the rate of appointment booking and the rate of patient engagement differed significantly between these two groups.

We also performed a campaign-level analysis. Each call record was tagged with its campaign (purpose) code, enabling grouping of outcomes by campaign type. We examined selected high-volume campaigns individually (“outpatient follow-up - hospital,” “no-show rescheduling,” “breast cancer screening outreach,” “abnormal result follow-up,” etc.) to understand variations in performance. We calculated crude and adjusted booking rates for each campaign and tabulated the distribution of outcomes (booked, already had, declined, no response) for representative preventive versus safety follow-up campaigns. A chi-square test was conducted to test for overall heterogeneity in engagement outcomes across all campaign types.

All statistical analyses were conducted using IBM SPSS Statistics Version 28 (IBM Corp., Armonk, NY). Given the very large sample size, a significance level of p<0.05 was used, with the understanding that even small differences may become statistically significant. Because this was a real-world quality improvement project rather than a controlled experiment, we interpret any observed differences as associations. We did not calculate confidence intervals for the large N percentages, as the margins of error would be very small (<0.2% for most outcomes given N>500,000).

Ethical considerations

This study was undertaken as a quality improvement initiative aimed at enhancing routine patient care. Institutional review board (IRB) approval was not required according to local policy, as the activities were intended for internal quality improvement and did not constitute human subjects research. No patient-identifiable data are presented in this report; all analyses were conducted on de-identified, aggregated operational data. Throughout the study, care was taken to ensure patient privacy (e.g., call outcomes were stored on secure internal systems). The decision to publish the findings was made in the interest of sharing learning, in line with SQUIRE guidelines, and all data are reported at a summary level only.

## Results

Overall outreach activity and primary outcome

A total of 548,089 outbound call attempts were completed during the evaluation period. The primary outcome, successful appointment booking, occurred in 66,238 (12.1%) calls when calculated using total call attempts as the denominator.

Operational exclusions included 3,241 (0.6%) calls due to incorrect contact information and 591 (0.1%) calls involving deceased patients. Patients reporting an existing upcoming appointment accounted for 90,774 (16.6%) calls. After excluding these non-actionable contacts, 453,483 calls were considered operationally eligible for appointment activation, yielding an adjusted booking rate of 66,238 (14.6%) calls.

Among all call attempts, declined scheduling was recorded in 235,012 (42.9%) calls, no response or voicemail in 141,173 (25.7%) calls, and lack of available appointment slots in 3,307 (0.6%) calls.

The distribution of all outreach outcomes across total call attempts is summarized in Table 1, which provides a process-level overview of outreach dispositions using total calls as the denominator.

**Table 1:** Distribution of final dispositions for all outbound outreach call attempts conducted during the evaluation period. Percentages are calculated using total call attempts as the denominator. “Already had appointment” indicates patients who reported an existing scheduled follow-up at the time of outreach. “Wrong number” and “deceased outcomes” represent operational exclusions and were excluded from adjusted analyses.

Outcome Category	Calls, n (%)
Booked appointment	66,238 (12.1)
Already had appointment	90,774 (16.6)
Declined	235,012 (42.9)
No response/voicemail	141,173 (25.7)
No slot available	3,307 (0.6)
Wrong number	3,241 (0.6)
Deceased	591 (0.1)
Total call attempts	548,089 (100)

Outcomes by care setting

After excluding operationally ineligible contacts, 406,485 hospital-based outreach attempts and 141,604 satellite clinic attempts were eligible for booking analysis. Successful appointment booking occurred in 50,154/406,485 (12.3%) hospital-based calls and 16,084/141,604 (11.4%) satellite clinic calls, representing a statistically significant but small absolute difference between care settings (χ² = 94.84, p < 0.001).

For engagement analyses, patients reporting existing follow-up arrangements were retained, consistent with the a priori definition of engagement. Using this broader denominator, engagement - defined as either appointment booking or confirmation of existing follow-up - occurred in 128,537/415,251 (31.0%) hospital-based calls compared with 28,475/137,617 (20.7%) satellite clinic calls (χ² = 1,238.6, p < 0.001). Given the large sample size, statistically significant findings reflect directional operational differences rather than causal effects (Table 2).

**Table 2:** Appointment booking and engagement outcomes by care setting following centralized outreach. Booking analysis excludes operationally ineligible contacts (wrong numbers, deceased patients) and is restricted to calls in which appointment scheduling was possible. Engagement analysis includes patients who reported existing follow-up arrangements, as engagement reflects continuity of care rather than booking conversion alone. Engagement was defined a priori as appointment booking or confirmation of an existing follow-up plan; non-engagement included declined scheduling or no response/voicemail. Percentages are calculated using the valid call denominator for each analysis.

Care Setting	Booking Analysis		Engagement Analysis		
	Valid calls for booking (n)	Booked appointments, n (%)	Valid calls for engagement (n)	Engaged, n (%)	Non-engaged, n (%)
Main hospital	406,485	50,154 (12.3)	415,251	128,537 (31.0)	286,714 (69.0)
Satellite clinics	141,604	16,084 (11.4)	137,617	28,475 (20.7)	109,142 (79.3)

Campaign-level outcomes

Table 3 presents booking outcomes across selected high-volume outreach campaigns. Crude booking is reported as the proportion of all outreach attempts resulting in a newly scheduled appointment. To account for patients who already had follow-up arranged at the time of contact, adjusted booking is also reported. Adjusted booking reflects the proportion of booked appointments among calls eligible for scheduling, defined as outreach attempts excluding patients who reported an existing appointment.

**Table 3:** Campaign-level appointment booking outcomes. Outcomes are reported as n (%). “Booked” represents newly scheduled appointments resulting from outreach. “Already had appointment” indicates confirmation of a pre-existing follow-up arrangement at the time of contact. “Adjusted booked” represents the proportion of booked appointments among calls eligible for scheduling, calculated after excluding patients who reported already having follow-up arranged. Percentages for adjusted booking use “Already had appointment” as the denominator.

Campaign	Setting	Calls, n	Booked, n (%)	Already Had Appointment, n (%)	Adjusted Booked, n (%)
OPD follow-up	Hospital	133,071	27,193 (20.4)	23,726 (17.8)	27,193 (24.9)
Cancelled	Hospital	70,609	9,684 (13.7)	27,094 (38.4)	9,684 (22.3)
No-show	Hospital	57,166	8,913 (15.6)	8,762 (15.3)	8,913 (18.4)
OPD follow-up	Satellite	29,029	8,310 (28.6)	3,580 (12.3)	8,310 (32.7)
Abnormal results follow-up	Hospital	16,349	4,182 (25.6)	5,116 (31.3)	4,182 (37.2)
Breast cancer screening	Hospital	13,849	671 (4.8)	213 (1.5)	671 (4.9)
Discharged inpatient	Hospital	10,164	1,284 (12.6)	1,624 (16.0)	1,284 (15.0)

Across campaigns, adjusted booking rates were consistently higher than crude booking rates, reflecting the smaller eligible denominator after exclusion of pre-arranged follow-up. Administrative recovery campaigns, including OPD follow-up and no-show outreach, demonstrated moderate adjusted booking yields, whereas abnormal results follow-up showed the highest adjusted booking proportion. Preventive screening campaigns, particularly breast cancer screening, demonstrated the lowest adjusted booking despite exclusion of patients with existing appointments.

These results describe variation in appointment activation across campaign types and reflect differences in outreach context and eligibility rather than comparative effectiveness.

Preventive and safety campaigns

Table 4 summarizes outcome distributions for preventive and safety-focused outreach campaigns, presenting adjusted booking rates alongside disaggregated engagement outcomes (already arranged care, declined, and no response). These campaigns are reported separately because their primary objective extends beyond appointment activation to include verification of continuity of care and mitigation of clinical risk in populations where perceived urgency and follow-up behavior vary substantially.

**Table 4:** Preventive and safety outreach outcomes by campaign type. Outcomes are reported as n (%), with percentages calculated using the total number of outreach attempts per campaign as the denominator. “Adjusted booking” reflects new appointment scheduling following exclusion of operationally ineligible contacts. “Already had follow-up” indicates confirmation of an existing appointment at the time of outreach. “Declined outreach” reflects successful contact without appointment activation. “No response/voicemail” indicates unsuccessful contact.

Campaign	Outreach Attempts, n	Adjusted Booking, n (%)	Already Had Follow-Up, n (%)	Declined Outreach, n (%)	No Response/Voicemail, n (%)
Breast cancer screening	13,849	734 (5.3)	197 (1.4)	6,760 (48.8)	4,848 (35.0)
Abnormal results follow-up	16,349	4,232 (25.9)	5,098 (31.2)	5,414 (33.1)	2,705 (16.5)
Colon cancer screening	7,611	1,218 (16.0)	1,362 (17.9)	3,136 (41.2)	1,621 (21.3)
Discharged inpatient follow-up	10,164	1,810 (17.8)	5,854 (57.6)	1,199 (11.8)	834 (8.2)
Discharged emergency department	11,611	1,707 (14.7)	4,193 (36.1)	3,796 (32.7)	1,846 (15.9)

Across campaigns, adjusted booking rates varied widely, reflecting differences in clinical intent and patient readiness rather than outreach execution alone. Preventive initiatives (e.g., cancer screening) were characterized by lower immediate booking rates and higher proportions of declined or non-response outcomes, whereas safety-focused campaigns (e.g., abnormal results and post-discharge follow-up) demonstrated higher levels of existing care continuity and overall engagement. These patterns highlight heterogeneity in outreach outcomes driven by baseline clinical context and follow-up expectations.

Table 5 evaluates whether patient engagement outcomes were uniformly distributed across outreach campaigns. A chi-square test demonstrated significant heterogeneity in engagement rates by campaign type (χ² = 62,785.5, df = 134, p < 0.001), indicating that observed differences in engagement were unlikely to be due to random variation. Given the large sample size and the diversity of campaign intents and target populations, these findings reflect substantial operational and contextual variation in patient response patterns across campaigns rather than comparative effectiveness. Table 5 summarizes engagement and non-engagement outcomes across outreach campaigns and presents the results.

**Table 5:** Heterogeneity of engagement outcomes across outreach campaigns. Engagement was defined as appointment booking or confirmation of an existing follow-up arrangement. Non-engagement included declined outreach or no response/voicemail. The chi-square test assesses whether engagement outcomes are uniformly distributed across outreach campaigns.

Comparison	Test	χ²	df	p-value
Engagement vs non-engagement across campaigns	Chi-square	62,785.5	134	<0.001

## Discussion

This system-wide quality improvement initiative demonstrates that a centralized outbound telephone outreach program can meaningfully support appointment activation and continuity of care across a multi-center healthcare network. Over seven months, more than half a million outreach attempts resulted in both new appointment scheduling and confirmation of existing follow-up, indicating that centralized outreach functions not only as a scheduling mechanism but also as a method for detecting and closing gaps in care engagement. Similar large-scale outreach evaluations have shown that proactive contact improves care continuity when implemented as part of routine operations rather than isolated pilot programs [3,10].

The observed appointment booking rates align with contemporary evidence supporting live telephone outreach. A systematic review by Opon et al. reported that predictive, phone-based reminder strategies were associated with a relative reduction in no-show rates of approximately 39% compared with usual care, particularly in high-risk populations [10]. In addition, a randomized quality improvement trial by Tarabichi et al. demonstrated that augmenting automated reminders with live telephone calls significantly reduced no-show rates and narrowed racial disparities in appointment adherence, highlighting the added value of human outreach beyond automation alone [3]. Our findings extend this evidence by demonstrating feasibility and impact at system scale across multiple care settings and campaign intents.

Engagement outcomes varied significantly by care setting, with higher engagement observed in the tertiary hospital compared with satellite clinics. Stronger discharge planning, clearer follow-up pathways, and higher perceived clinical urgency in hospital-based care are likely associated with improved follow-up adherence.

Campaign-level heterogeneity was pronounced. Preventive screening campaigns demonstrated the lowest immediate booking rates and the highest proportions of declined or non-response outcomes. This pattern is consistent with evidence showing that preventive outreach is limited by behavioral and perceptual barriers rather than access alone [7]. Randomized trials and population-based studies have reported modest uptake from telephone or mail outreach for cancer screening, even when interventions are effective relative to usual care [11,12]. In contrast, safety-focused outreach, such as abnormal result follow-up and post-discharge contact, demonstrated higher engagement and confirmation of existing follow-up, reflecting greater perceived urgency and alignment with patient expectations, as described in diagnostic safety and transitional care literature [13].

Importantly, these findings support the use of engagement-based metrics rather than booking alone when evaluating outreach programs. Recent patient safety frameworks emphasize closed-loop communication, defined as not only initiating follow-up but also verifying completion or documenting patient disengagement [13]. Studies have shown that failures in follow-up communication contribute substantially to diagnostic delay and preventable harm, underscoring the safety value of outreach programs that capture confirmation of existing care arrangements [6,13].

Operationally, a substantial proportion of outreach attempts resulted in declined scheduling or unsuccessful contact. Similar patterns have been reported in other large outreach initiatives and are frequently attributed to outdated contact information, competing patient priorities, and limited perceived benefit [7,10]. Evidence suggests that multimodal strategies combining live calls with SMS reminders, predictive risk stratification, and navigation support may improve reach and efficiency, particularly for patients with higher barriers to engagement [3,14].

This evaluation has limitations. As a retrospective observational analysis without a control group, causal inference is not possible. Analyses were conducted at the call-attempt level, which may overrepresent patients requiring repeated contact and limit the independence of observations. In addition, downstream outcomes such as appointment attendance, clinical outcomes, and patient-reported experience were not assessed. These limitations are consistent with real-world quality improvement evaluations and have been widely acknowledged in similar system-level studies [13].

Despite these limitations, the findings demonstrate that centralized outreach can be implemented at scale, produce actionable operational insights, and support continuity of care across diverse clinical contexts. Centralized models may improve standardization, equity, and visibility of follow-up gaps compared with fragmented, clinic-based approaches. Future work should evaluate downstream clinical outcomes, incorporate predictive analytics, and assess the cost-effectiveness of hybrid outreach models integrating automation with targeted human engagement.

## Conclusions

A centralized outbound call program in a multi-center healthcare system substantially improved care continuity, scheduling more than 66,000 appointments and confirming follow-up for approximately 90,000 additional patients over seven months. Effectiveness varied by setting and campaign type, with a stronger impact in post-acute and abnormal-result follow-up than in preventive outreach. This pragmatic quality improvement initiative demonstrates that centralized outreach is feasible at scale and can be integrated into routine operations. Despite the absence of a control group, the level of engagement observed suggests meaningful operational benefit. Future work should focus on targeted, multi-modal strategies and on measuring downstream clinical and utilization outcomes to better define system-wide value.
